# Address to Our Readers

**Published:** 1850-01-01

**Authors:** 


### ADDRESS TO OUR READERS.

---

WITH the present number of our Journal we enter upon the *third* year of our eventful existence. We have outlived a most unfavourable prognosis. Dismal were the forebodings with which our entrance into life was greeted. Persons who wished us a happy and prosperous voyage, speculated (as kind friends often do) either upon our gradual decay or premature dissolution. Thank God, we still breathe. We can throw ourselves into the editorial chair, and with philosophical benignity, look back with pleasure upon the *past*, and with glowing anticipations towards the *future*. We do not wish our kind friends to misunderstand us. We have not for the last two years been reposing upon a bed of down. Our editorial life has not been one of ease and quiet. Our path has not been across flowery meads, and by the side of purling streams. The road we have travelled has been a rugged and circuitous one, often full of great discouragement, and as we have paused on our journey, we have occasionally allowed the thought to glance through our mind, whether we should ever reach the goal towards which we were bending our weary steps.

The idea of establishing a Psychological Journal in this country was one of our early aspirations. Twenty years before the first number issued from the press, we had sketched in MS. the design of the work! Our notion was anticipated by the production, in Paris, of the *Annales Médico-Psychologiques;* and in New York by the American *Journal of Insanity.* This circumstance did not alter our intentions, and we resolved, on the first favourable opportunity, upon launching our fragile bark upon

"———Those stormy seas,"

often said to be

"Bankrupt of life and prodigal of ease."

Conscious that we were engaged in the advocacy of a good cause, we felt that if we merited it, we should receive encouragement and support.

Our readers will kindly bear in remembrance the facts, that, to a certain extent, we had to create a taste for medico-psychological speculations—that our Journal was confined to a *spécialité*, and addressed itself to a small class of readers.

Since its publication, we have been extremely gratified to perceive that our humble efforts to place on its proper basis the science of medico-psychology, has given an impetus to such inquiries. Many of our contemporaries have devoted portions of their respective journals to this subject, and a few public lecturers have particularly referred to the matter, as one deserving of more attention than it has hitherto received in this country.

Much of our success is to be attributed to the kind support which has so generously been extended towards us by contemporary journals. To them we owe a deep debt of gratitude, which it will never be in our power to repay. Appreciating the difficulties surrounding our novel undertaking, and foreseeing the dark clouds which were destined to hover about our path, we heard from them words of hope, praise, kindness, and encouragement. We desire to express most emphatically our lasting obligations to those gentlemen who extended towards us so friendly a hand, and who cheered us on in the exercise of our responsible and anxious duties.

Although we have reason to believe that the *Journal of Psychological Medicine* has become a portion of the *established* periodical literature of the country, we do not wish our kind friends and subscribers to relax in their efforts to promote its circulation in quarters where it has not yet penetrated.

It has required no ordinary effort on our part to obtain for this Review its present *status*, and we trust by the kind exertions of our friends it may yet take a higher position.

45, *Albemarle Street*, *Piccadilly*.

